# Comparison of smoking conventional cigarettes and using heated tobacco products on the olfactory and gustatory function in healthy young adults: A cross-sectional study

**DOI:** 10.18332/tid/192524

**Published:** 2024-09-11

**Authors:** Ella Sever, Daniela Kovačević Pavičić, Aleksandar Pupovac, Ema Saltović, Stjepan Špalj, Irena Glažar

**Affiliations:** 1 Clinic of Dental Medicine, Clinical Hospital Center Rijeka, Rijeka, Croatia; 2 Department of Prosthodontics, Faculty of Dental Medicine, University of Rijeka, Rijeka, Croatia; 3 Department of Dental Medicine, Faculty of Dental Medicine and Health, Josip Juraj Strossmayer University of Osijek, Osijek, Croatia; 4 Department of Periodontology, Faculty of Dental Medicine, University of Rijeka, Rijeka, Croatia; 5 Department of Orthodontics, Faculty of Dental Medicine, University of Rijeka, Rijeka, Croatia; 6 Department of Oral Medicine, Faculty of Dental Medicine, University of Rijeka, Rijeka, Croatia

**Keywords:** smoking, taste, tobacco products, smell

## Abstract

**INTRODUCTION:**

Smoking has a negative impact on the chemosensory function. The aim of the study was to evaluate the effects of smoking experience and type of tobacco products on gustatory and olfactory function.

**METHODS:**

This study included 30 conventional cigarette smokers, 30 heated tobacco products (HTPs) users, and 30 non-smokers. Olfactory function was assessed with a ‘Sniffin' Sticks Screening 12 Test’ while the taste function was assessed with ‘taste strips’ for the basic tastes of sweet, sour, salty, and bitter. The lifetime exposure to smoking was calculated using the Brinkman index (BI).

**RESULTS:**

Conventional cigarette smokers demonstrated decreased olfactory function in comparison to non-smokers (median: 10 vs 11; p=0.001) but HTPs users did not differ between those two groups. Overall gustatory function was lower in conventional cigarette smokers (median: 9.5) and HTPs users (median: 10) than in non-smokers (median: 14; p<0.001). A difference was detected in the sour, salty and bitter taste but without significant difference between HTPs users and conventional cigarette smokers. Negative linear correlations were found between the BI and olfactory function, overall gustatory function, sour, salty, and bitter taste (r= -0.317 – -0.585; p≤0.002). In multiple linear regression, BI was the only predictor of olfactory dysfunction when controlling for the effect of tobacco products, age, and gender accounting for 11% of variance (p=0.024, R^2^=0.123). For gustatory dysfunction, BI was the strongest predictor followed by gender and tobacco products accounting for 11%, 5%, and 4% (p<0.001, R^2^=0.259).

**CONCLUSIONS:**

Olfactory and gustatory function are adversely associated with smoking, more depending on BI than tobacco product.

## INTRODUCTION

The chemosensory systems enable us to perceive both odors and flavors. These interconnected systems oversee the perception of flavors and aromas, and also serve as warning signs that protect us from dangerous situations^1^. The prevalence of olfactory and gustatory dysfunction varies in a population^2^. There are multiple causes of smell and taste disorders, with the most important ones being ageing, respiratory infections, head trauma, vitamin deficiency, medications, neoplasia, or environmental factors^1,2^. The most common environmental factor is associated with smoking^1^. Conventional cigarettes burn tobacco and produce smoke that contains a mixture of potentially harmful and harmful constituents such as carcinogenic molecules, carbon monoxide, nicotine, heavy metals, and other irritants. When the olfactory and gustatory systems are exposed to these harmful constituents, it can lead to sensory impairment^2,3^.

Alternative smoking devices such as the heated tobacco products (HTPs) have been developed. HTPs provide a different mechanism that produces lower temperatures, thus reducing the number of harmful and potentially harmful constituents^3,4^. Despite being relatively new, these products have gained popularity among young adults^5^. Studies have shown that using HTPs may lead to the initiation of smoking among non-smokers and cause former smokers to relapse^6,7^. The majority of research is centered on the relationship between conventional cigarette smoking and taste and smell disturbances. However, there is limited evidence investigating the association between smell and taste disturbances and the use of HTPs.

Cigarette smoke has an impact on the respiratory tract, with inflammation and mutagenic effects being the most common outcomes. Some of its components also damage olfactory epithelium causing injury or death of cells^1,8^. These harmful constituents in tobacco smoke can cause squamous metaplasia in which normal columnar olfactory epithelium is replaced by squamous epithelium. Smokers also have an increased risk of reversible sinonasal inflammation and inflammation of the olfactory mucosa, which is associated with decreased olfactory function^9^. The effect of smoke can also indirectly increase susceptibility to other factors that can harm the olfactory mucosa through an inflammatory process^10^. According to several studies, smoking can also affect olfactory function centrally. The exposure of olfactory tissue to cigarette smoke leads to a reduction in the capacity to produce sensory cells, resulting in a loss of olfactory recognition^11^. In addition, there is evidence that smokers have significantly smaller olfactory bulbs and less grey matter volume in the olfactory gyrus in comparison with non-smoker^10^.

Smoking has both direct and indirect negative effects on gustatory function. Increased exposure to tobacco and cigarette smoke is associated with a decrease in the number of taste-associated structures and morphological changes to them^12^. Furthermore, there is evidence that long-term smoking can lower salivary flow rate and cause hyposalivation, with downstream effects on gustatory function. Chronic smoking is also connected to lower levels of important nutrients (such as vitamin B, E, zinc, and folic acid), all of which play crucial roles in affecting taste perception^3^. On the central level, nicotine influences the taste signal by acting on the central nervous system. It has been shown that the application of nicotine on the tongue modifies the response of the neurons in the nucleus of the solitary tract of rats^13^.

In this study, we aim to evaluate and compare the effects of HTPs and conventional cigarettes on olfactory and gustatory function.

## METHODS

### Study design and population

This stratified cross-sectional study was conducted at the Clinic of Dental Medicine, Clinical Hospital Center, Rijeka, Croatia, and the Faculty of Dental Medicine University of Rijeka, Croatia. Participants (n=90) were recruited from December 2021 to December 2023, categorized into three groups: HTPs users (n=30), conventional cigarette smokers (n=30), and non-smokers (n=30).

The inclusion criteria for the smokers’ group were: aged ≥18 years, a positive smoking history, and an absence of systemic and local diseases that can affect chemosensory function (listed below). Participants were classified as conventional cigarette smokers if they had smoked at least 100 cigarettes after starting smoking. HTPs users were identified if they had used at least 100 HEETs and had not smoked any other types of cigarettes in the past six months. The lifetime exposure to smoking was calculated using the Brinkman index (BI), which is calculated by multiplying daily consumption by years of smoking^14^. The inclusion criteria for the non-smokers’ group were the same as for the smokers’ group, except for a history of not smoking.

The exclusion criteria included the use of other forms of tobacco, dual users (current, simultaneous use of conventional cigarettes and HTPs products), history of alcohol consumption, participants with a history of upper respiratory infections within the past three weeks, sinonasal disorders (nasal polyps, chronic rhinosinusitis, allergic rhinitis, severe septum deviation), a history of exposure to respiratory toxins other than cigarettes, malignancy, head trauma, neurologic, endocrine, and psychiatric disorders.

### Questionnaire

All participants were required to fill in a questionnaire regarding their overall health and smoking habits. The questions covered general information, details about smoking (such as average number of cigarettes smoked per day and duration of smoking in years), information about any systemic or local diseases, and prescribed medications.

### Olfactory testing

Olfactory function testing was performed with a validated ‘Sniffin’ Sticks Screening 12 Test’ (Burghart GmbH, Wedel, Germany). All ‘Sniffin’ Sticks’ tests were performed by oral medicine resident (ES). During the test, participants were asked to identify the odors using pen-like odor dispensing devices. For each of the 12 sticks, the pen’s cap was removed and the tip of the pen was placed approximately 1–2 cm in front of the nose for approximately three seconds. The participant had to choose the correct odor from a list of four words indicating different odors. The test performance was based on the number of correct answers with scores ranging 0 to 12; scores 0–6 indicated anosmia, 7–10 indicated hyposmia, and 11–12 indicated normosmia^15^.

### Gustatory testing

The gustatory testing was conducted using a validated ‘taste strips’ test, which is used for the detection and quantification of taste disorders. In addition, all ‘taste strips’ tests were performed by oral medicine resident (ES). Taste function was assessed for four basic tastes: sweet, sour, salty, and bitter. One hour before testing, subjects were asked not to eat or drink anything except water. The 16 taste strips were presented in a pseudo-randomized sequence, using a whole-mouth procedure in the middle of the anterior portion of the tongue. Additionally, two blank strips were presented. Participants were asked to identify the taste quality choosing one of five possible answers on a form (sweet, sour, salty, bitter, no taste). Before the assessment of each taste strip, the mouth was rinsed with water. Test performance was based on the number of correct answers, whereby the overall taste performance and the performance of each taste quality were evaluated separately. For the overall taste performance scores ranged 0–16. Scores below the threshold value of 9 were considered hypogeusia, and failure to detect the highest concentrations of all four taste qualities was considered complete ageusia^16^.

### Statistical analysis

Statistical analysis was performed using the Statistical Package for the Social Sciences (SPSS), version 13.0 (SPSS, Inc., Chicago, IL, US). The normality of distribution of variables was tested with the Kolmogorov-Smirnov test. Variables that did not have normal distribution were expressed as median and interquartile range. Categorical variables were expressed as frequencies and percentages. The comparison of the general differences between the evaluated groups were made by Kruskal-Wallis test for continuous variables and chi-squared test for the qualitative variables. Mann-Whitney test was used as a *post hoc* test after Kruskal-Wallis, while z-test for proportions as *post hoc* after chi-squared with Bonferroni correction for multiple comparisons in both situations. Spearman correlation was used to assess the relation between BI and the indices of olfactory, gustatory function, and each of the four basic tastes. To identify factors of importance for olfactory and gustatory dysfunction, a multiple linear regression was used. Multivariate model was built on biological plausibility. The following explanatory variables were selected in the model: age, gender, tobacco products, and BI. The essential assumptions for regression were tested: normality of residuals (probability-to-probability plot), linearity (scatter plot of relationship between BI and chemosensory function), homoscedasticity (scatter plot of residuals), and the absence of multicollinearity (correlation coefficients between predictors and variance inflation factor). Unstandardized (B) and standardized coefficients (β) were calculated. The unstandardized coefficient represents the change in the dependent variable associated with a one-unit change in the corresponding independent variable, while holding other variables constant. The standardized coefficient expresses the average change in standard deviations of a dependent variable associated with a one standard deviation change in an independent variable. Zero order, partial and part correlations are also presented. Zero order defines correlation between dependent and independent variable without controlling the influence of other variables. Partial correlation is the association after controlling for the influence of other variables on both the independent and dependent variable. Interaction of predictors was also tested in multivariate regression models. For the part correlation only of the control variables on the independent is taken into account and it presents the unique contribution of the independent variable. All tests were two-tailed and statistical significance was accepted at the p<0.05 level.

## RESULTS

### Demographic characteristics of study population

The participants’ age ranged from 20 to 55 years (median: 32 years, IQR: 25–43), with 67% being female (Table 1). The study comprised three groups, each with 30 participants, and no statistically significant differences were observed between the groups in terms of age and sex. In the group of HTPs users, the median number used per day was 12, with a median duration of 6 years. For the conventional cigarette smokers, the median number of cigarettes smoked per day was 12, with a median smoking duration of 10 years. BI was lower for the HTPs users compared to the conventional cigarette smokers (73.5 vs 112.5), but the difference did not reach statistical significance (Table 1).

**Table 1:** Sociodemographic, smoking data and chemosensory prevalence of the participants among non-smokers, HTPs users and conventional cigarette smokers, cross-sectional study conducted at the Clinic of Dental Medicine, Clinical Hospital Center, Rijeka, Croatia, and Faculty of Dental Medicine, University of Rijeka, Croatia, December 2021 to December 2023 (N=90)

*Characteristics*	*Non-smokers (N=30) n (%)*	*HTPs users (N=30) n (%)*	*Conventional cigarette smokers (N=30) n (%)*	*p*
**Age** (years)				
Median (IQR)	31 (24.75–40.75)	32.5 (25–42)	32.5 (25.25–43.75)	0.973^§^
Range	20–55	21–55	20–55	
**Gender**				
Male	10 (33)	10 (33)	10 (33)	1.000^†^
Female	20 (67)	20 (67)	20 (67)	
**Brinkman index**				
Median (IQR)	0	73.5 (31.25–100)	112.5 (50–240)	0.061^§^
Range	0	5–280	20–600	
**Olfactory dysfunction**				
Yes	1 (3)^a^	6 (20)^a,b^	11 (37)^b^	0.005^†^
No	29 (97)	24 (80)	19 (63)	
**Gustatory dysfunction**				
Yes	3 (10)^b^	14 (47)^a^	15 (50)^a^	0.002^†^
No	27 (90)	16 (53)	15 (50)	

§ Kruskal Wallis test.

† Chi-squared test with *post hoc* z-test with Bonferroni correction for multiple comparisons. Groups that share the same superscript letters do not differ significantly.

IQR: interquartile range.

### Olfactory and gustatory dysfunction

**Figure 1:**
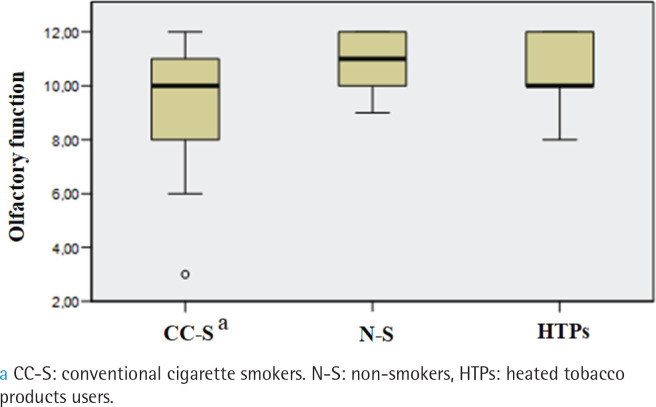
Comparison of olfactory function between conventional cigarette smokers, non-smokers and HTPs users, cross-sectional study conducted at the Clinic of Dental Medicine, Clinical Hospital Center, Rijeka, Croatia, and Faculty of Dental Medicine, University of Rijeka, Croatia, from December 2021 to December 2023 (N=90)

Prevalence of hyposmia differed between groups (p=0.005). It was found in 20% of HTPs users, 37% of conventional cigarette smokers, and 3% of non-smokers, with significant differences between cigarette smokers and non-smokers, but HTPs users did not differ from the other groups. No subjects had anosmia. Olfactory function significantly differed between the three groups (p<0.001). The conventional cigarette smokers demonstrated decreased olfactory function in comparison to non-smokers (median: 10, IQR: 8–11 vs median: 11, IQR: 10–12; p=0.001) while HTPs users did not differ from conventional cigarette smokers and non-smokers (median: 10, IQR: 10–12) (Figure 1).

**Figure 2:**
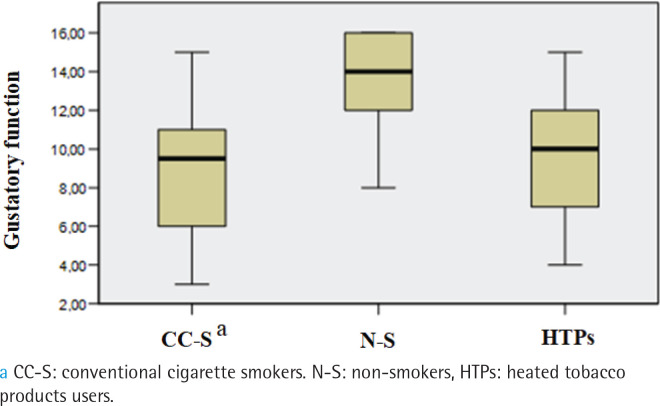
Comparison of overall gustatory function between conventional cigarette smokers, non-smokers and HTPs users, cross-sectional study conducted at the Clinic of Dental Medicine, Clinical Hospital Center, Rijeka, Croatia, and Faculty of Dental Medicine, University of Rijeka, Croatia, from December 2021 to December 2023 (N=90)

**Figure 3:**
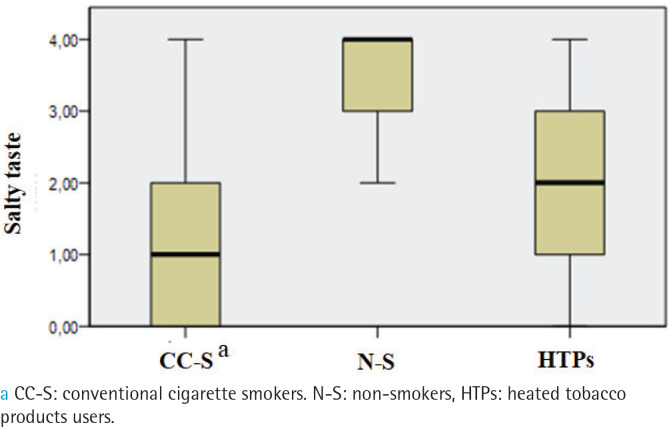
Comparison of salty taste between conventional cigarette smokers, non-smokers and HTPs users, cross-sectional study conducted at the Clinic of Dental Medicine, Clinical Hospital Center, Rijeka, Croatia, and Faculty of Dental Medicine, University of Rijeka, Croatia, from December 2021 to December 2023 (N=90)

**Figure 4:**
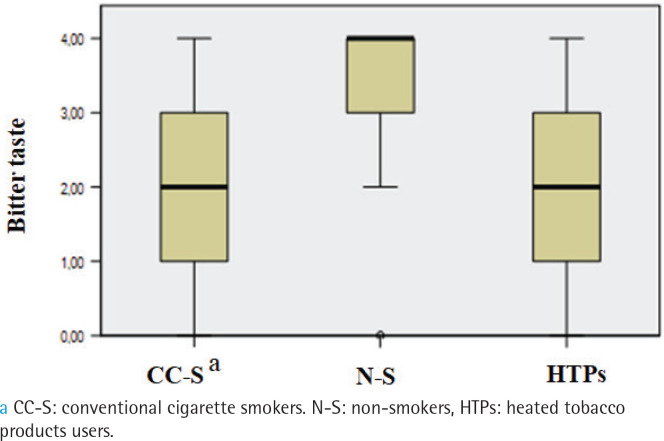
Comparison of bitter taste between conventional cigarette smokers, non-smokers and HTPs users, cross-sectional study conducted at the Clinic of Dental Medicine, Clinical Hospital Center, Rijeka, Croatia, and Faculty of Dental Medicine, University of Rijeka, Croatia, from December 2021 to December 2023 (N=90)

The prevalence of hypogeusia differed between groups (p=0.002). It was observed in 47% of HTPs users, 50% of conventional cigarette smokers, and 10% of non-smokers, with non-smokers significantly differing from the other two groups, while no differences were found between the smokers and the HTPs users. No subjects were found to have ageusia. Overall gustatory function significantly differed between the three groups (p<0.001), being lower in conventional cigarette smokers (median: 9.5) and HTPs users (median: 10) than non-smokers (median:14; p<0.001) (Figure 2). The salty taste was significantly higher in non-smokers (median: 4) than both HTPs users (median: 2) and conventional cigarette smokers (median: 1; p<0.001) (Figure 3). Smoker and HTPs users did not differ significantly. Similar results were found for bitter (median: 4 vs 2 vs 2; p<0.001) (Figure 4) and sour (median: 4 vs 2 vs 2; p<0001) (Supplementary file Figure 1). Sweet taste was the same in all three groups (median: 4) (Supplementary file Figure 2).

In the whole sample, the BI was significantly linearly negatively correlated with gustatory (r= -0.548; p<0.001) and olfactory function (r= -0.317; p=0.002), and the correlation was weak for smell and moderate for taste. As the smoking index increases, the chemosensory function decreases. The smoking index was most closely related to the taste of salty (r = -0.585; p<0.001), followed by bitter (r= -0.448; p<0.001), and sour (r=0.482; p<0.001). The correlation coefficient for sweet did not exceed 0.25, which was considered a significant association. Age did not correlate with chemosensory function nor with the BI.

Gender influenced gustatory but not olfactory function. Hypogeusia was more prevalent in males (19/29; 66%) than females (13/61; 21%; p<0.001). Males had lower gustatory function score than females (median: 8 vs 12; p=0.010), which was seen in sweet (3 vs 4; p<0.001) and bitter taste (2 vs 3; p=0.025). BI was not related to gender.

Multiple linear regression models were used to explore predictors of chemosensory dysfunction. The independent variables considered in the analysis were gender, age, type of tobacco product, and smoking index. The only predictor of olfactory dysfunction was the smoking index (p=0.001) when the influence of the type of tobacco product, age, and gender was controlled, and accounted for 11% of the variance (p=0.024, R^2^=0.123; Table 2). Increase in BI for 1 scalar point decreased the olfactory function score by 0.004 scalar points (95% CI: -0.007 – -0.002). No interaction of predictors was detected. For taste dysfunction, the smoking index was the most significant predictor (p=0.001), followed by gender (p=0.016) and type of tobacco product (HTPs) (p=0.039), whose unique contribution accounted for 11%, 5%, and 4% variance, respectively (p<0.001, R^2^=0.259) (Table 2). Increase in BI for 1 scalar point decreased the taste function score by 0.009 scalar points (95% CI: -0.015 – -0.005), female gender increased the taste function by 1.8 scalar points (95% CI: 0.3–3.2) while HTPs decrease it by 1.5 (95% CI: -2.8–0.1). No interaction of predictors was detected.

**Table 2:** Multiple linear regression models for the prediction of olfactory and gustatory dysfunction, cross-sectional study conducted at the Clinic of Dental Medicine, Clinical Hospital Center, Rijeka, Croatia, and Faculty of Dental Medicine, University of Rijeka, Croatia, from December 2021 to December 2023 (N=60)

*Variables*	*Unstandardized coefficients*			*Standardized coefficients*	*p*	*Correlations*		
	*B*	*SE*	*95% CI*	*β*		*Zero-order*	*Partial*	*Part*
Olfactory dysfunction*								
**Constant**	10.4	0.9						
**Age** (years)	0	0	-0.0–0.1	0.2	0.101	0.061	0.177	0.169
**Brinkman index**	0	0	-0.0 – -0.0	-0.4	**0.001**	-0.273	-0.338	-0.336
**Gender** (1=Male; 2=Female)	-0.4	0.4	-1.1–0.3	-0.1	0.253	-0.068	-0.124	-0.117
**HTPs** (0=no; 1=yes)	0.2	0.4	-0.5–0.9	0.1	0.547	0.048	0.066	0.061
Gustatory dysfunction**								
**Constant**	9	1.7						
**Age** (years)	0	0	-0.1–0.1	0	0.877	-0.155	0.017	0.014
**Brinkman index**	0	0	0.0 – -0.0	-0.4	**0.001**	-0.413	-0.361	-0.333
**Gender** (1=Male; 2=Female)	1.8	0.7	0.3–3.2	0.2	**0.016**	0.304	0.259	0.23
**HTPs** (0=yes; 1=no)	-1.4	0.7	-2.8–0.1	-0.2	**0.038**	-0.197	-0.223	-0.197

* R=0.350; R^2^=0.123; adjusted R^2^=0.082; p=0.024.

** R=0.509; R^2^=0.259; adjusted R^2^=0.224; p<0.001.

## DISCUSSION

This study demonstrated that olfactory and gustatory function is altered in consumers of conventional cigarettes and HTPs products, and influenced by lifetime exposure to smoking. According to the literature, current smokers – whether they smoked conventional cigarettes or use other forms of tobacco – were at increased risk for olfactory and gustatory dysfunction. To date, there are no clinical studies corresponding to the chemosensory dysfunction effects of this new tobacco product.

Conventional cigarette smoking has detrimental effects on olfactory function. A large systemic review and meta-analysis demonstrated that current smokers have approximately 60% increased risk of olfactory dysfunction compared to non-smokers^10^. Our findings are generally consistent with previous research that supports the association between conventional cigarette smoking and decreased smell function. According to our model, the main predictor of olfactory dysfunction is smoking experience, and it was not influenced by gender, age, and HTPs use. Several studies that have assessed smell function by measuring odor threshold, odor discrimination, and odor identification via the ‘Sniffin’ Sticks’ test found that conventional cigarette smokers had lower thresholds, identification, discrimination, and overall scores compared to non-smokers^17,18^. The present study contributes to the existing literature by confirming that smoking conventional cigarettes is a potential risk factor for olfactory dysfunction. Additionally, it suggests that new HTPs products might have fewer deleterious effects on olfactory function, which can be explained by the reduction of harmful and potentially harmful constituents in the tobacco aerosol. On the contrary, two studies that applied only an identification test failed to find the relationship between smoking and smell impairment^19,20^. In our study, we also used a screening identification test, which may explain why our HTPs users did not show a difference compared to non-smokers. Several studies have also reported a negative correlation between the intensity of smoking and reduced olfactory function. The greater the number of cigarettes or packs smoked per day, the more diminished the olfactory perception becomes^2,18^. A large population-based study in Germany evaluated 1277 individuals using the same ‘Sniffin’ Sticks Screening 12’ identification test and found the highest prevalence of hyposmia in smokers and a positive dose-response relationship between the daily consumption of cigarettes and hyposmia^2^. According to the research, olfactory dysfunction is more pronounced in current smokers, men, and older people^2,11,20^. To our knowledge, the presence of smell disturbance in HTPs users has not been previously studied. However, the results of the 2021 National Health Interview Survey (NHIS) indicate that e-cigarette users exhibited the highest prevalence of a smell disorder among non-tobacco users, conventional cigarette users, e-cigarette users, and poly-tobacco product users^12^. In comparison, Larsen et al.^20^ measured odor identification ability in chronic smokers (n=135) using 16 odor items and reported that smell function in smokers did not vary significantly by age, race, or gender.

Significant taste impairment and gustatory dysfunction in conventional cigarette smokers have been previously observed and explained in several studies^3,21-23^. Our findings indicate that conventional cigarette smokers and HTPs users have decreased gustatory function, without significant difference between these two groups. Several studies suggest that smokers have a lower sensitivity for certain tastes in comparison to non-smokers^9,21,23^. According to the nationally representative data in the US, chronic smokers with higher nicotine dependence experience decreased intensity of bitter and salty tastes^12^. However, other studies did not report significant differences between certain tastants, even though some reported elevated taste perception in smokers^24,25^. Our findings support the conclusion that smokers, in general, have depressed sensitivity for sour, bitter, and salty taste. Interestingly, HTPs users demonstrated better taste ability than conventional cigarette smokers, particularly in detecting salty tastes. This improved salty taste sensitivity in the HTPs group may also be explained by the reduction of harmful chemicals. Another possible explanation is that our study included mostly younger participants with shorter smoking histories. Similar to the olfactory function, a negative dose-response relationship was found between the number of cigarettes or packs smoked per day and gustatory function^2,22^. In a study by Khan et al.^22^, a negative linear correlation was found between intensity of smoking and threshold sensitivity. A similar relationship was found in our research, where an increase in the duration and intensity of smoking led to a lower sensitivity for overall taste sensitivity and certain tastes (sour, salty, bitter). This relationship could be explained by the harmful effects of tobacco smoke and consequently a decrease in the number and changes in the structure of fungiform papillae on the tongue’s surface^3^. According to the 2021 National Health Interview Survey (NHIS), taste disorders were more prevalent in women, the elderly, and users of poly-tobacco products^12^. This study showed that gustatory perception depends on the smoking experience, gender, and type of tobacco product. Although the prevalence of taste impairment is higher in older people, age was not found to be an important predictor in our study. Also, contrary to NHIS, taste was better in females in our study. The type of tobacco products is an important factor that suggests HTPs can also cause decrease gustatory perception^12^. This is a first study evaluating effects of HTPs on gustatory function. Despite the findings in the literature that suggest that these new tobacco products are less harmful than conventional cigarettes, our research suggests otherwise, indicating similar effects to those seen in conventional cigarette smokers.

### Limitations

The main limitation of the study is a relatively small sample size and the fact that the study included mainly younger participants with a shorter smoking history. Although we looked for other potential causes of taste and smell impairment, we could not be certain that we did not overlook other reasons. Additionally, we assessed individual health and systemic comorbidities using a questionnaire, which could lead to false results. Another limitation arises from the cross-sectional study design, which does not allow for establishing a time sequence between smoking and smell and taste impairment. Assessment of olfactory function was performed with the screening ‘Sniffin’ Sticks’ test, therefore it might have been less precise than gustatory testing. We only looked at current smoking habits and did not take into account past usage history in both heated tobacco product (HTP) users and non-smokers, which could also impact the sense of smell and taste. Furthermore, smoking status was evaluated with a questionnaire and the Brinkman index was used to assess the relationship between smoking intensity and its effects on chemosensory function. Having more detailed and objective smoking data, such as cotinine levels, would allow for a more comprehensive analysis. Further research with larger sample sizes is needed to understand the potential side effects and risks of smoking on the sense of taste and smell.

## CONCLUSIONS

Olfactory and gustatory function are adversely associated with smoking, more depending on BI than tobacco product.

## Supplementary Material



## Data Availability

The data supporting this research are available from the authors on reasonable request.
