# Supplementary material for: Comparison of smoking conventional cigarettes and using heated tobacco products on the olfactory and gustatory function in healthy young adults: A cross-sectional study
Source: Tob Induc Dis. 2024 Sep 11;22:10.18332/tid/192524. doi: 10.18332/tid/192524 (PMC11389167; doi:10.18332/tid/192524)
Supplement: Supplementary file 1 [file TID-22-156-s1.pdf]

Supplementary Figure 1. Comparison of sour taste between conventional cigarette smokers, nonsmokers and HTPs users, cross-sectional study conducted at the Clinic of Dental Medicine, Clinical Hospital Center, Rijeka, Croatia, and Faculty of Dental Medicine, University of Rijeka, Croatia, from December 2021 to December 2023 (N=90)

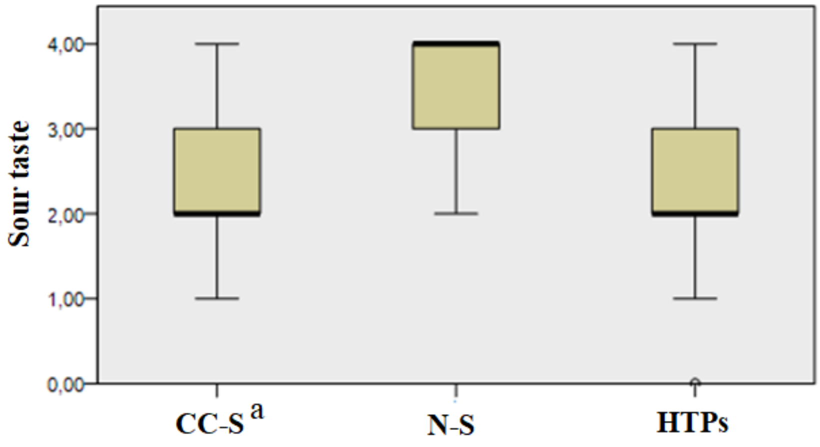

[a] CC-S conventional cigarette smokers, N-S nonsmokers, HTPs Heated tobacco products smokers.

Supplementary Figure 2. Comparison of sweet taste between conventional cigarette smokers, nonsmokers and HTPs users, cross-sectional study conducted at the Clinic of Dental Medicine, Clinical Hospital Center, Rijeka, Croatia, and Faculty of Dental Medicine, University of Rijeka, Croatia, from December 2021 to December 2023 (N=90)

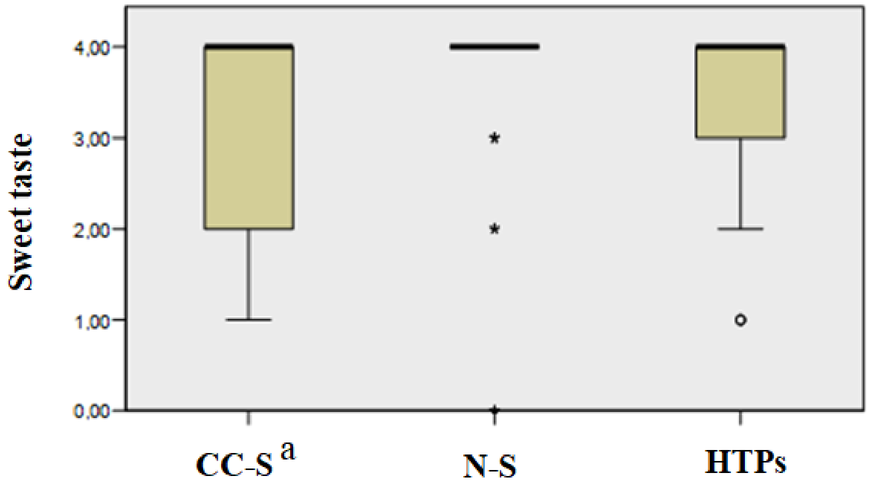

[a] N-S nonsmokers,HTPs Heated tobacco products smokers, CC-S conventional cigarette smokers.

© 2024 Sever E. et al.
